# Neuro-Oncologic Veterinary Trial for the Clinical Transfer of Microbeam Radiation Therapy: Acute to Subacute Radiotolerance after Brain Tumor Irradiation in Pet Dogs

**DOI:** 10.3390/cancers16152701

**Published:** 2024-07-29

**Authors:** Laura Eling, Samy Kefs, Sarvenaz Keshmiri, Jacques Balosso, Susan Calvet, Gabriel Chamel, Renaud Drevon-Gaud, Isabelle Flandin, Maxime Gaudin, Lucile Giraud, Jean Albert Laissue, Paolo Pellicioli, Camille Verry, Jean-François Adam, Raphaël Serduc

**Affiliations:** 1 Institut National de la Santé et de la Recherche Médicale UA7 Synchrotron Radiation for Biomedicine, Université Grenoble Alpes, 38400 Saint-Martin-d’Hères, France; sarvenaz.keshmiri@univ-grenoble-alpes.fr (S.K.); jean-francois.adam@univ-grenoble-alpes.fr (J.-F.A.); raphael.serduc@inserm.fr (R.S.); 2 Centre Hospitalier Universitaire Grenoble Alpes, Maquis du Grésivaudan, 38700 La Tronche, France; samy.kefs@inserm.fr (S.K.); jbalosso@chu-grenoble.fr (J.B.); iflandin@chu-grenoble.fr (I.F.); cverry@chu-grenoble.fr (C.V.); 3 Argos Clinique Vétérinaire Pierre du Terrail, 38530 Pontcharra, France; sue.calvet@pierreduterrail.vet; 4 Clinical Oncology Unit, Small Animal Internal Medicine Department, University of Lyon, VetAgro Sup Campus Vétérinaire, 69280 Marcy l’Etoile, France; gabriel.chamel@vetagro-sup.fr; 5 Unité de Recherche Interaction Cellules Environnement, University of Lyon, VetAgro Sup Campus Vétérinaire, 69280 Marcy l’Etoile, France; 6 ARGOS, Clinique Vétérinaire de l’Esplanade, 38000 Grenoble, France; renaud.drevon.gaud@wanadoo.fr; 7 OnlyVet, Centre Hospitalier Vétérinaire, 69800 Saint Priest, France; m.gaudin@onlyvet.fr (M.G.); l.giraud@onlyvet.fr (L.G.); 8 Pathology Institute of Bern, University of Bern, 3012 Bern, Switzerland; jean-albert.laissue@unibe.ch; 9 European Synchrotron Radiation Facility, 38000 Grenoble, France; paolo.pellicioli@unibe.ch

**Keywords:** Synchrotron Microbeam Radiation Therapy, dog brain tumor, acute to subacute radiation effects, clinical transfer phase

## Abstract

**Simple Summary:**

The benefit of spatially fractionated radiotherapy for brain tumors is maximized through Synchrotron Microbeam Radiation Therapy (MRT). In 2021, the clinical transfer phase of MRT began: the first brain-tumor-bearing dog patients were treated under clinical conditions in view of the forthcoming clinical transfer. As a primary endpoint, the tolerance of normal brain tissues to MRT was evaluated, while the efficacy in reducing tumor volume was considered as a secondary endpoint. We here present acute to subacute neurologic radiotolerance and tumor volume reduction after MRT for brain tumor treatment in canine patients included in our ongoing veterinary trial, proving that MRT is a safe tool for spontaneous brain tumor treatment in dogs.

**Abstract:**

Synchrotron Microbeam Radiation Therapy (MRT) has repeatedly proven its superiority compared with conventional radiotherapy for glioma control in preclinical research. The clinical transfer phase of MRT has recently gained momentum; seven dogs with suspected glioma were treated under clinical conditions to determine the feasibility and safety of MRT. We administered a single fraction of 3D-conformal, image-guided MRT. Ultra-high-dose rate synchrotron X-ray microbeams (50 µm-wide, 400 µm-spaced) were delivered through five conformal irradiation ports. The PTV received ~25 Gy peak dose (within microbeams) per port, corresponding to a minimal cumulated valley dose (diffusing between microbeams) of 2.8 Gy. The dogs underwent clinical and MRI follow-up, and owner evaluations. One dog was lost to follow-up. Clinical exams of the remaining six dogs during the first 3 months did not indicate radiotoxicity induced by MRT. Quality of life improved from 7.3/10 [±0.7] to 8.9/10 [±0.3]. Tumor-induced seizure activity decreased significantly. A significant tumor volume reduction of 69% [±6%] was reached 3 months after MRT. Our study is the first neuro-oncologic veterinary trial of 3D-conformal Synchrotron MRT and reveals that MRT does not induce acute to subacute radiotoxicity in normal brain tissues. MRT improves quality of life and leads to remarkable tumor volume reduction despite low valley dose delivery. This trial is an essential step towards the forthcoming clinical application of MRT against deep-seated human brain tumors.

## 1. Introduction

To date, the diagnosis of brain cancer is invariably associated with a grave prognosis due to the radiosensitivity of normal brain tissues, which limits effective tumor control by radiotherapy. Very few therapeutic improvements have emerged in recent decades [1]. This is true not only for brain cancer in human patients but also in dogs. Pet owners often decline radiotherapy because of its poor geographic availability and its high cost. To optimize the current radiotherapy protocols, dogs bearing spontaneously occurring brain tumors are perfectly adapted research subjects due to their close pathophysiological, anatomical, and clinical resemblance to human patients [2]. In return, preclinical trials can provide therapeutic benefits for canine patients.

Spatially fractionated radiation therapy (SFRT) has long-established advantages in sparing normal tissues while efficiently ablating tumor tissues [3]. For example, GRID radiotherapy, invented in the beginning of the 20th century [4], later led to the uncovering of the so-called “dose-volume effect” on the micrometer scale [5]. Indeed, Microbeam Radiation Therapy (MRT) fully exploits the benefits of SFRT. MRT is currently best performed with synchrotron-generated orthovoltage X-rays, collimated into an array of 50 micron-wide, 400 micron-spaced beamlets, by delivering hundreds of Grays in the microbeam path (peak dose), at ultra-high dose rates (several thousands of Grays per second). Conversely, the diffusing inter-beam valley dose amounts to only a few rays. This highly heterogeneous dose distribution provides an excellent radiation tolerance of normal tissues in the brain [6], in well-vascularized organs [7] and in immature tissues [8]. In contrast, a preferential brain tumor-killing effect, presumably due to the disruption of neoplastic vessels, asphyxia and tumor necrosis [9], occurred in preclinical rodent gliosarcoma models after MRT [10]. Importantly, MRT has repeatedly provided more efficient tumor control and caused less normal tissue damage in rodent brains than homogenous “broad” beam exposures [11]. 

Using multiple irradiation ports has recently shown to improve the therapeutic results of MRT on 9L rat gliomas. Indeed, five MRT ports, delivering a cumulated valley dose of 10 Gy, are as effective, in terms of animal survival and tumor control, as a homogeneous dose delivering 25 Gy [12]. The same five-port protocol has been safely tested on normal rat brains [12] and on normal brains of Yucatan minipigs (to be published), without eliciting cognitive deficits. It has already been shown that MRT can be delivered as a 3D-conformal, deep-targeting radiosurgical tool in large animals [13]. Some questions remain before passing on to the first prospective human patient MRT trial. We seek answers to them in our ongoing veterinary trial. 

In 2021, the first pet dog suffering from glioma was treated using multidirectional, 3D-conformal MRT as proof of principle of microbeam safety and of clinical patient workflow [14]. We now present the first results of the complete group, i.e., seven dogs exposed to a 2.8 Gy minimal cumulated MRT valley dose, delivered to the tumor through five irradiation ports.

## 2. Materials and Methods

### 2.1. Canine Patient Inclusion

**Figure 1:**
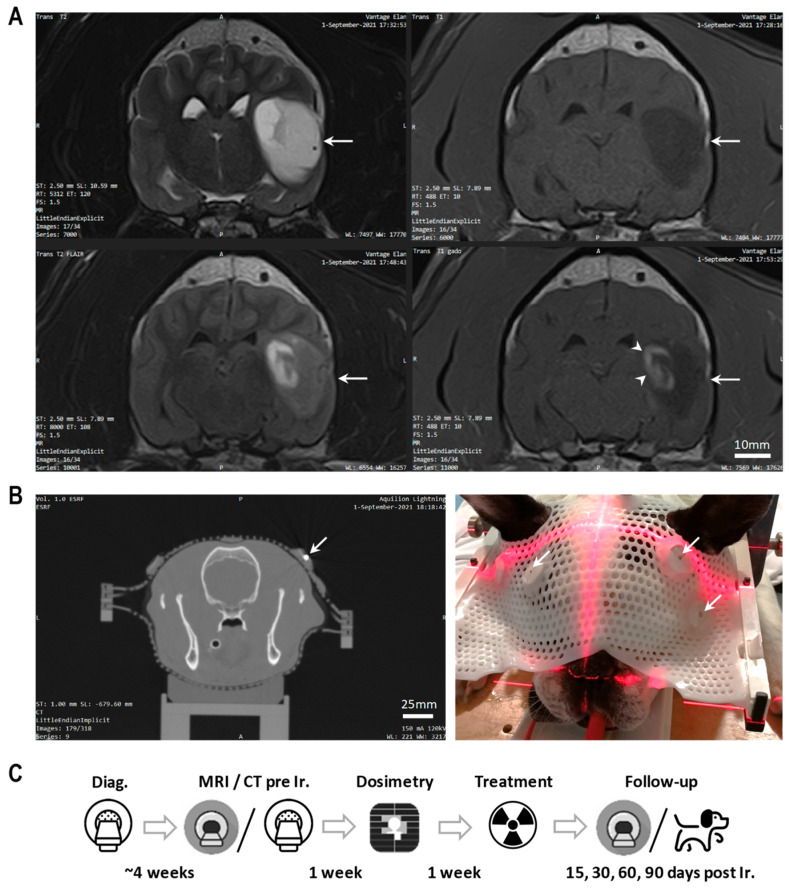
MRI, CT scan and workflow for Microbeam Radiation Therapy in dog patients. (**A**) Magnetic resonance images of suspected glioma in a dog before the MRT fraction (**upper left**: Trans T2 [hyperintense], **upper right**: Trans T1 [hypointense], **lower left**: Trans T2 FLAIR [heterogeneous], **lower right**: Trans T1 Gd-DOTA). Detection of an intra-axial, extra-ventricular heterogenous lesion (arrow), situated in the left temporal lobe and showing partial contrast enhancement (arrowhead). The lesion measured 25 mm in diameter and induced compression of the left lateral ventricle and deviation of the midline. (**B**) Dosimetric CT scan (**left**) and photo (**right**) of the same animal, including the thermoplastic mask and metallic fiducial markers (arrows). (**C**) Workflow from diagnosis to animal follow-up.

Dogs with a presumptive diagnosis of glioma, consistent with their medical history, breed, sex, weight, age, and clinical signs [15], were considered for inclusion and were referred to the same veterinary hospital (OnlyVet, Saint-Priest, France) for diagnostic MRI. The following characteristics on MR images were required (see Figure 1A): the presence of a single intra-axial but extra-ventricular lesion, with associated mass effect, T2-hyperintense, T1-hypointense, FLAIR-heterogeneous, and T1 contrast enhancement (partial or complete) after gadolinium (Gd-DOTA) injection [16]. In accordance with previous data [17], the mass could be associated with hemorrhage, cyst-like structures and peritumoral edema. The lesion had to be <33 mm in diameter due to physical beam constraints. We did not perform a biopsy as per standard practice. However, all MR images were reviewed by a European College of Veterinary Neurology board-certified specialist, and by the deputy director of neuroradiology and MRI at the university hospital. We recorded the dose and therapeutic scheme of supportive medical treatments (phenobarbital, levetiracetam, corticosteroids) before and after MRT exposure. Regular dosage of phenobarbital plasma concentration was performed. We excluded dogs (1) diagnosed with another malignant tumor within 6 months before the irradiation, (2) having a history of inflammatory, vascular or traumatic CNS disease, (3) presenting any other potentially lethal pathology, or (4) requiring immunomodulatory, cytotoxic or anti-angiogenic medication. To identify comorbidities, all dogs had a complete blood count, serum biochemical analysis, and thoracic and abdominal CT scan performed prior to the cranial radiotherapy. Before inclusion in the study, we informed the dogs’ owners about the potential risks related to radiation exposure and asked them to sign a consent form. The study was approved by the Ethical Committee of VetAgro Sup, Veterinary University of Lyon, France (project number 2248V2). The study design, including its objectives, the inclusion and exclusion criteria, all mandatory exams, the conduct and planification of the study, the medical care, and the imaging protocol, was written down in an underlying protocol prior to the study onset.

### 2.2. Treatment Planning, Dosimetry and Microbeam Radiation Therapy

For detailed information on treatment planning, dosimetry, patient positioning and the MRT exposure, please refer to [14] and to Appendix A. Briefly, a volumetric dosimetry CT scan served for preparation of the image-guided MRT positioning, using a patient-specific thermoplastic mask (Brainlab, Germany) and fiducial markers (see Figure 1B). Dose calculations for five coplanar and conformal irradiation ports were carried out using the MRT-specific dose algorithm [18]. A minimal cumulated valley dose of 2.8 Gy was prescribed to the planning target volume (PTV) (4 Gy in 70% of the PTV (PTV70)), corresponding to 20–25 Gy peak dose per port. Each dog was exposed to image-guided, single-fraction, Synchrotron MRT (50 µm width, 400 µm spacing, 121 keV mean energy, 5500 Gy/s dose rate). A clinically designed patient safety system assured a secure radiation exposure. The scan and irradiation were carried out under general anesthesia (ketamine/xylazine 5:1 mg/kg i.v. followed by isoflurane inhalation 2.5% in 1 L air/min). 

### 2.3. Clinical Follow-Up after MRT

**Figure 2:**
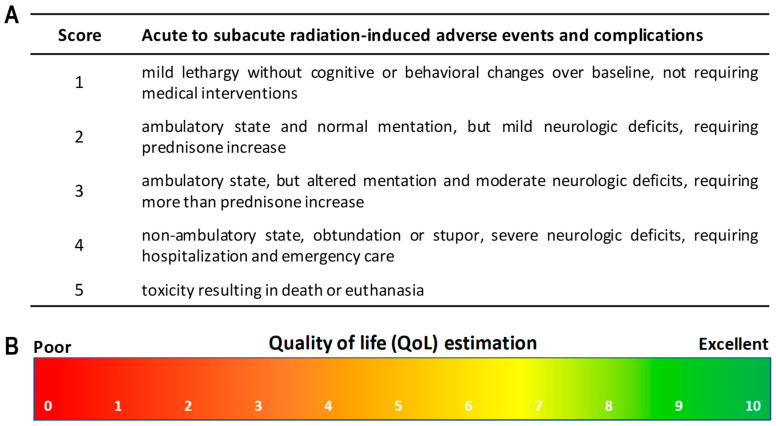
Clinical scoring scheme and QoL scheme. (**A**) Clinical scoring scheme for veterinary evaluations after the MRT exposure, compared with the baseline health status. Adapted from [19,20]. (**B**) QoL estimation scheme for owner evaluations before and after the treatment.

The referring veterinarians performed a complete general and neurological physical examination before MRT and 15, 30, 60, and 90 days (written as Tn) after irradiation (see workflow in Figure 1C). At the same time points, the owners answered standardized questionnaires. The patients’ general health and behavior were tightly monitored throughout the entire study period, including seizure number and severity (non-convulsive vs. convulsive seizures). We evaluated radiation toxicity in accordance with the veterinarian scoring scheme for acute to subacute adverse events and complications of the VRTOG v1.0 and v2.0 guidelines [19,20]. Scores ranged from 1 (no change over baseline) to 5 (death or euthanasia); these are summarized in Figure 2A. Figure 2B shows the quality of life (QoL) evaluation scheme on a scale from 0 (poor QoL) to 10 (excellent QoL), estimated by the owners. For statistical analysis of convulsive versus non-convulsive seizure number, we used a 2-way analysis of variance (significant for *p* < 0.05), with either Bonferroni’s multiple comparisons test (conv. vs. non-conv.) or Turkey’s multiple comparisons test (comparing the 6 time points) in GraphPad Prism. For statistical analysis of the total seizure number at the different time points, we used parametric, paired *t*-tests (significant for *p* < 0.05) in GraphPad Prism. 

### 2.4. MR Imaging before and after MRT

All dogs underwent initial MRI before irradiation (T0) and MRI controls 30 days (T30) and 90 days (T90) after the treatment (T2 and T1 Gd-DOTA-injected sequences for tumor volume analysis using the MicroDicom software, MicroDicom DICOM Viewer 2024.2 ×86). We evaluated the tumor volume, the relative tumor volume evolution of T0–T30 and T0–T90, and the tumor response of the individual patients at T30 and T90 (including partial tumor response if the tumor volume decreased by ≥30% from the baseline). Statistical analysis was performed with GraphPad Prism using parametric, paired *t*-tests (significant for *p* < 0.05).

## 3. Results

### 3.1. Dog Patient Inclusion and Medical Treatment Combined with MRT

**Figure 3:**
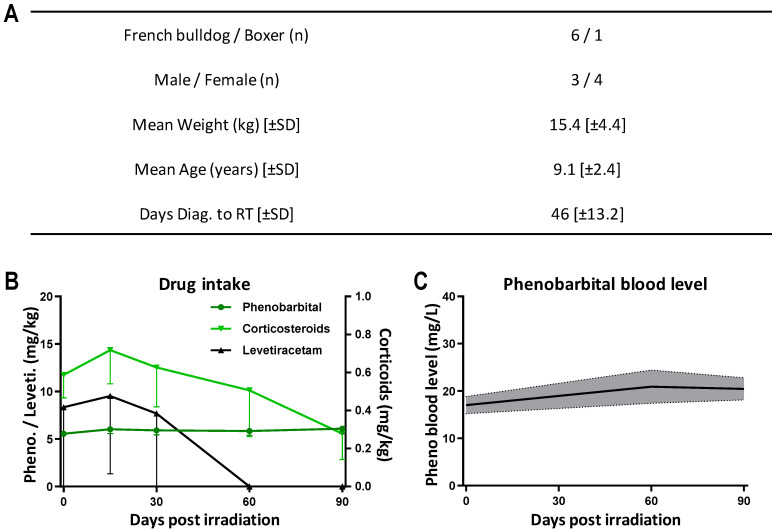
Dog patient inclusion and medical treatment. (**A**) Dog patient inclusion regarding breed, sex, weight, age and the days elapsed from diagnosis to MRT. (**B**) Drug intake (mg/kg/d): phenobarbital and levetiracetam plotted on the left Y-axis, corticosteroids plotted on the right Y-axis. Data are plotted as mean ± SEM. (**C**) Phenobarbital blood level (mg/L). Data are plotted as mean ± SEM.

Seven pet dogs met the inclusion criteria. We successfully exposed these canine patients to MRT. One dog, lost to follow-up, was excluded from the data analysis. We analyzed data from five French bulldogs and one boxer (see Figure 3A), of which three dogs were males and three dogs were females. The mean weight was 15.4 [±4.4] kg and the mean age was 9.1 [±2.4] years. Tumor-induced seizure activity started, on average, 46 [±13.2] days before the radiation exposure. Details on the supportive medication are depicted in Figure 3B. All dogs received phenobarbital (5–6 mg/kg/d) and corticosteroids (prednisolone 0.6 mg/kg/d). Two dogs received additional levetiracetam doses (60 mg/kg/d) before and/or in the first few days after the MRT exposure, which was then tapered within 1 month post irradiation. The doses of corticosteroids, which were slightly increased in two dogs in the first few days post MRT, were reduced and discontinued starting from 2 weeks after the exposure. The phenobarbital dose was tailored to ensure that serum levels remained within the therapeutic range of 15 to 35 mg/L (Figure 3C). 

### 3.2. No Acute to Subacute Radiotoxicity after MRT

**Figure 4:**
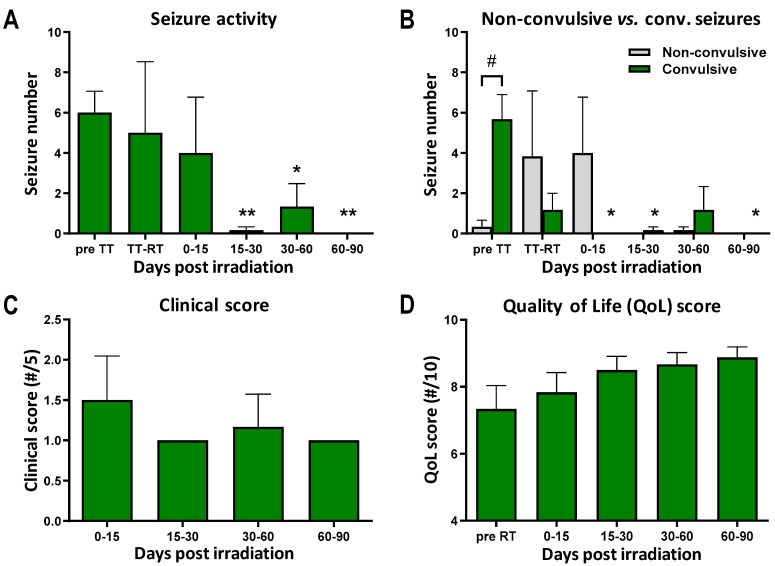
Clinical follow-up and quality of life. (**A**) Seizure activity (number/d). Data are plotted as mean ± SEM and significance was determined using parametric, paired *t*-test: * for *p* < 0.05 versus pre treatment (TT), ** for *p* < 0.005 versus pre TT. Pre TT: period before drug intake; TT-RT: period from start of drug intake to radiotherapy; Pre RT: period before radiotherapy. (**B**) Non-convulsive versus convulsive (conv.) seizures (number/d). Data are plotted as mean ± SEM, and significance was determined using 2-way ANOVA for *p* < 0.05: * Tn (time at n days post RT) versus pre TT, # convulsive versus non-convulsive seizures. (**C**) Clinical score (from 0 to 5). Data are plotted as mean ± SEM. (**D**) QoL score (from 0 to 10). Data are plotted as mean ± SEM.

Figure 4A,B show that MRT reduced seizure activity, whereas seizing continued with medical treatment only. No convulsive seizures were induced in the acute phase after MRT. However, partial seizures were documented in three out of six dogs (4 [±2.8] non-convulsive seizures) on the first day post MRT, which were controlled by increasing the dose of prednisolone. A significant reduction in seizure frequency was observed from 2 weeks to 3 months post MRT (*p* < 0.005), compared with the initial seizure number (6 [±2.8] seizures before starting medical treatment); indeed, a near absence of seizures was achieved. Only one dog had noteworthy convulsive seizures at around 50 days after the MRT exposure. No local skin toxicity or depilation in the irradiation field were detected. The clinical score, as judged by the veterinarians, did not worsen in the subacute post MRT period (see Figure 4C and Appendix B). It was the highest (1.5/5 [±0.2]) in the immediate post MRT phase due to slight lethargy and the presence of partial seizures. Consistently, quality of life (QoL) improved after MRT, as estimated by the owners; the mean QoL score increased from 7.3/10 [±0.7] before MRT to 8.9/10 [±0.3] at 3 months after MRT (Figure 4D). 

### 3.3. Tumor Volume Reduction after MRT

**Figure 5:**
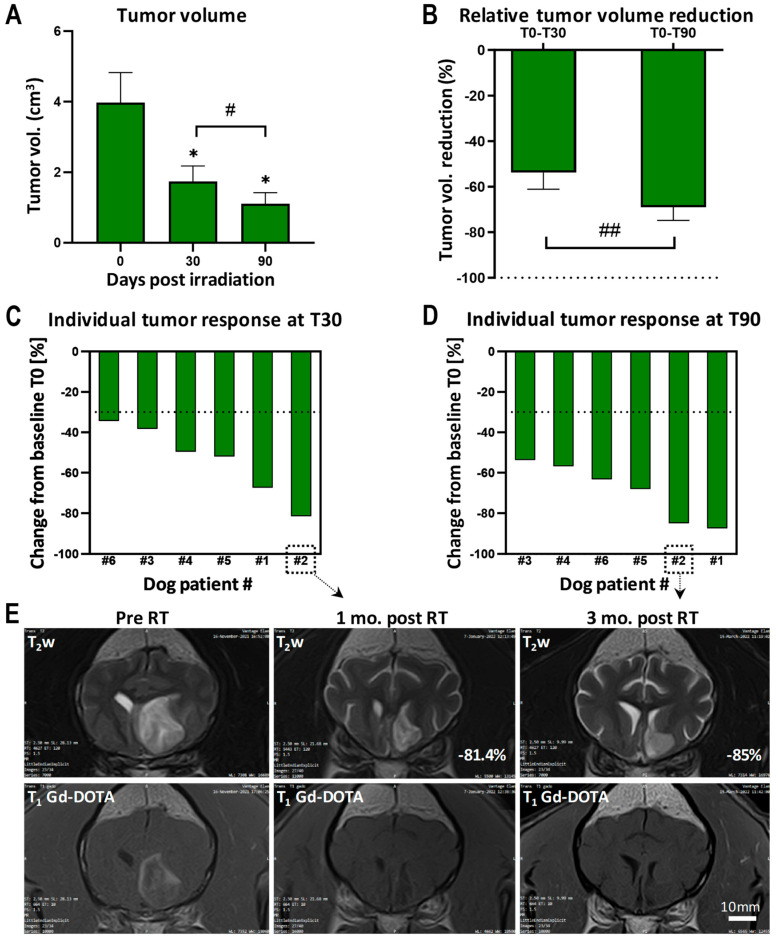
Tumor volume and its reduction. (**A**) Tumor volume (cm^3^). Data are plotted as mean ± SEM and significance was determined using parametric, paired *t*-test for *p* < 0.05: * Tn (time at n days post RT) versus pre RT, # T30 versus T90. (**B**) Relative tumor volume reduction (%). Data are plotted as mean ± SEM and significance was determined using parametric, paired *t*-test: ## for *p* < 0.01. (**C**) Individual tumor response at 30 days post MRT, compared with the baseline tumor volume at T0. (**D**) Individual tumor response at 90 days post MRT, compared with the baseline tumor volume at T0. The dashed line in (**C**,**D**) indicates a partial response (−30% decrease in the tumor volume). (**E**) Trans T2 images (**top row**) and Trans T1 images after gadolinium injection (**bottom row**) showing the intracranial mass of patient number 2 before the MRT exposure (**left**), at 1 month post MRT (**middle**) and at 3 months post MRT (**right**). Note the disappearance of contrast enhancement at 1 and 3 months after MRT.

The tumor volume was significantly decreased at 1 and 3 months after MRT, compared with the initial tumor volume (*p* < 0.05). The tumor volume was also significantly smaller at 3 months than at 1 month after MRT (*p* < 0.05). Tumors had shrunk to a mean volume of only 1.1 [±0.3] cm^3^ at 3 months (Figure 5A). A significant difference was also seen in the reduction in the relative tumor volume (Figure 5B): tumors were on average 53.8 [±7.3] % smaller 30 days after MRT than they were initially. The mean tumor volume reduction reached 69.0 [±5.8] % at 90 days post MRT (*p* < 0.01 for T0–T30 versus T0–T90). The tumor response of the individual canine patients is shown in Figure 5C (at 1 month post MRT) and 5D (at 3 months post MRT), compared with the baseline tumor volume at T0. All tumors responded to the treatment with a decrease of >30% at both time points. Representative MR images showing the tumor of dog patient number 2 can be seen in Figure 5E. The reduced tumor volume and the disappearing mass effect (normalization of the ventricular volume, straightening of the midline) were visible on the T2-weighted images (top row). No contrast enhancement was seen after MRT in this patient, contrary to the partial gadolinium uptake at the diagnostic MRI (T1 sequences after Gd-DOTA injection on the bottom row).

## 4. Discussion

This paper is a proof of the success of Microbeam Radiation Therapy as a conformal, clinically adapted treatment for deep-seated, spontaneous brain tumors, most likely glioma, in canine patients. Subsequent to the treatment of our first canine patient, as published in 2022 [14], we have completed the cohort of dogs treated with a cumulated MRT valley dose of 4 Gy in PTV70 (70% of the PTV), delivered through five ports. Therefore, we claim the feasibility of MRT for dogs in clinical conditions, i.e., perfect treatment preparation, logistics, and follow-up, as well as the safety of MRT. Indeed, no signs of radiotoxicity manifested in the acute to subacute phase. Inconsequential partial seizures, which responded readily to an increase in the dose of prednisolone, appeared in the first 24 h post irradiation. They were due to a mild and transient cerebral edema, which is a common phenomenon after conventional RT exposures [21]. Follow-up MRI did not reveal lesions in normal brain tissues; to the contrary, the mass effect of the initial tumor volume (deviation of the midline, ventricular compression) was noticeably reduced. Indeed, our multiport irradiation protocol allowed consequential dose reduction per port: a valley dose of only ~1 Gy was delivered per port, minimizing normal tissue dose in non-crossing parts of each array. This procedure spared normal tissues from high-dose radiation damage, while MRT peak and valley doses accumulated in the tumor. In addition, no veterinary concern arose during the first three months post MRT. Quality of life (QoL) improved, as judged by the owners. Note that the QoL scores in the first 4 weeks post MRT were partially biased by the side effects of the drug intake (polyuria-polydipsia, polyphagia, incontinence). The scores improved as the tapering of the corticosteroid doses began, in line with the adjuvant medication protocol for RT in dogs [21,22]. Aside from these drug adverse effects, the owners expressed high satisfaction in regard to the outcome of the radiotherapy and the wellbeing of their pets. 

Former studies show the great interest of therapeutic ionizing irradiation for brain cancer in dogs. Compared with symptomatic management, surgery or chemotherapy, radiation therapy has proven to be the most effective and safest treatment modality [23]. In addition, the advantages of spatially fractionated RT have been accentuated in a recent pet dog trial, in which pathological complete remission of de novo brain tumors was reached by a single fraction of linac-generated minibeam irradiation (mean dose of 26 Gy, point doses of ~30 Gy) [24]. Reflecting these results, we found an extraordinary tumor volume reduction of nearly 70% three months after delivery of a rather low MRT valley dose, cumulating to only 4 Gy in PTV70. The corresponding minimum cumulated peak doses of 20 to 25 Gy per port have not led to detectable radiation-induced brain injury, despite dose hot spots reaching up to 196 Gy in beam-crossing areas in the tumor. These spike-like hot spots, along with the amount of high-dose microbeam trajectories, play a key role in the effectiveness of tumor control and are tightly linked to the prescription of number of irradiation ports [12]. An increase in the port number combined with a higher cumulated MRT valley dose will certainly lead to an even more efficient tumor control [25]. We have shown so far that a minimal cumulated MRT valley dose of 2.8 Gy (4 Gy in PTV70) and only ~20 Gy peak dose per port are not sufficient to ablate the tumor completely. A dose escalation study is in preparation, which is indispensable before advancing to a first clinical trial. Escalating dose delivery is crucial to fully exploit the tumor control capacity of MRT, which is in line with the unique peak-to-valley vascular and immunologic responses of MRT, whilst defining the maximal tolerable dose for human patient exposures.

Evaluation of high-dose radiation toxicity and long-term animal follow-up are extremely important for the clinical transfer of any new irradiation modality. In fact, a recent pet dog trial using FLASH electron exposures for the treatment of oral cavity cancer has shown an alarming normal tissue toxicity [26] that may substantially limit FLASH therapy. Doses >30 Gy delivered at ultra-high dose rate have led to osteoradionecrosis in several patients, beginning at 3 months post irradiation, despite good tumor response. Similar results were found after FLASH electron therapy for nasal carcinoma in cats. Severe adverse events occurred and led to the premature interruption of the trial [27]. So far, MRT, delivered at ultra-high (FLASH) dose rates, has not induced side effects in dogs during the acute to subacute phase. First results of long-term normal tissue responses will soon be available and may help to evaluate radiobiological effects of heterogeneous dose distribution combined with a potential FLASH effect.

Our study opens new horizons for future treatment strategies and developments in radiotherapy. Other clinical targets, such as breast cancer [28,29,30], squamous cell carcinoma [31], melanoma [32], drug-resistant epilepsy [33], and osteosarcoma, as reviewed in [34], can be addressed in upcoming dog trials using MRT. Our well-established dog irradiations may provide new opportunities not only for the research community and clinicians, but also for veterinarians and pet owners. 

The clinical transfer of MRT for human brain cancer is foreseen once the dog trial is completed. MRT can be proposed as an excellent tool in targeting aggressive parts or the resection site of primary glial tumors, or in treating localized glioma recurrence, with efforts already made towards personalized MRT protocols [35]. MRT will likely be delivered as a boost as part of a hypofractionated RT protocol [36]. Results of the current dog trial also allow to further refine the MRT protocol, e.g., by non-coplanar targeting and temporal dose fractionation [37]. The ongoing development and future commercialization of microbeam compact sources [38,39,40], validated in vitro [41] and in the first in vivo experiments [42,43], will make MRT much more accessible in a hospital environment. The transfer of MRT for clinical applications thus relies on the final results of the ongoing dog trial, in particular on the outcome of the dose escalation study. We are currently gathering and analyzing data on survival, histopathologic post mortem tumor grading, normal tissue morphology, MRI characteristics, and dose-volume histograms. For comparison, we intend to expose a second cohort of pet dogs to conventional RT at the university hospital. 

## 5. Conclusions

In conclusion, the work presented here proves that dogs suffering from glioma can be safely exposed to and treated by microbeam X-rays. For the first time, a cohort of seven pet dogs has been successfully irradiated with conformal MRT. No signs of radiotoxicity occurred within the first 3 months of follow-up. An impressive tumor volume reduction, achieved by low MRT valley doses, whilst maintaining an excellent quality of life in treated dogs, confirms the promise of MRT for human clinical trials.

## Figures and Tables

**Figure A1:**
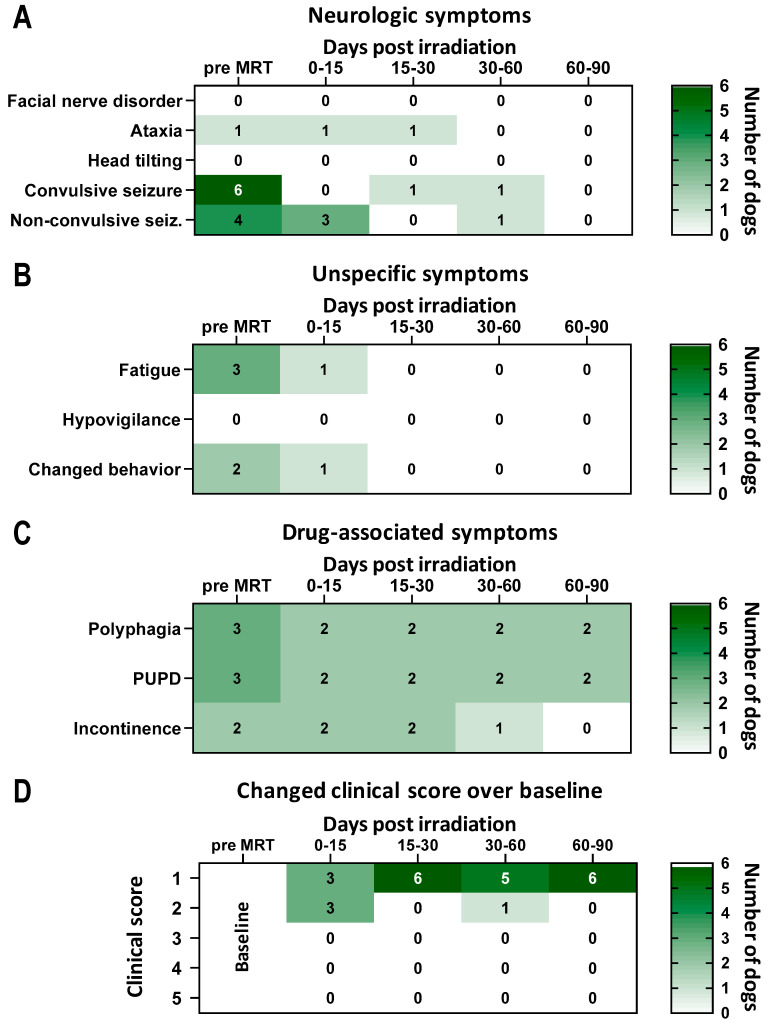
Veterinary evaluation of MRT-induced adverse events and complications. Upon veterinary exams before MRT (pre MRT: baseline) and at regular intervals post MRT (as a function of days post irradiation; columns), the number of dogs presenting (**A**) neurologic symptoms (rows), (**B**) unspecific symptoms (rows) and (**C**) drug-associated symptoms (rows) was noted. (**D**) At control visits, the clinical status was estimated, either as unchanged from baseline (1/5) or as deteriorated from baseline (2/5 to 5/5 depending on the symptom severity).

## Data Availability

Data are stored in an institutional repository and will be shared upon request to the corresponding author.

## Appendix A. Treatment Planning, Dosimetry and Microbeam Radiation Therapy

After the diagnosis of suspected glioma by MRI, all dogs underwent a volumetric CT scan; MR and CT images were merged for treatment planning. Contouring of the gross target volume (GTV), organs at risk (OARs), and positioning markers was carried out using the Isogray software (Dosisoft, France, https://www.dosisoft.com/, (accessed on 1 July 2024)). The irradiation dose was prescribed to the planning target volume (PTV = GTV + 1 mm). Two weeks after the dosimetric CT scan, MRT was carried out at the ID17 biomedical beamline of the ESRF. The dog patient was placed in a prone position on the goniometer with the head fixed in a thermoplastic mask. The homogeneous synchrotron X-ray beam was intensity modulated by means of a multislit collimator and filtered (Be: 2.3 mm, C: 1.13 mm, Al: 3.95 mm, Cu: 2.08 mm, Au: 0.0008 mm, and PMMA: 22 mm) to obtain a polychromatic energy spectrum in the range of 50–500 keV. Image-guided alignment of the treatment isocenter and the goniometer isocenter was absolved using synchrotron low-flux images (<50 mGy/image) [44]. The prescribed dose was delivered at ultra-high dose rate (5500 Gy/s) along the microbeam paths, and a custom-designed patient safety system assured the treatment safety as it would instantly stop the irradiation exposure within 10 ms if the incoming influx changed by more than 15%.

## Appendix B. Absence of Sub-/Acute MRT-Induced Adverse Events and Complications

Marking the baseline health state before MRT, all dogs presented neurologic symptoms, in particular convulsive seizures (see Figure A1A). One dog was ataxic, a symptom unrelated to the brain lesion but in line with a herniated disk (a common comorbidity in brachycephalic dogs). As pictured in Figure A1B, half of the animals showed increased fatigue related to the brain tumor and to the supportive drug treatment. The latter also induced typical changes in the eating and drinking behavior (Figure A1C), but it stabilized the general health condition and lowered the seizure activity.

Few changes over the baseline were induced by the MRT exposure. In the first 24 to 48 h post irradiation, few dogs were more tired than before the exposure or showed changed general behavior (less willing to play and walk). Apart from partial seizures occurring in 50% of the dogs, no new neurologic symptoms appeared. In these animals, the clinical score was estimated at 2/5 (Figure A1D). From 2 weeks onward, the general wellbeing of all dogs was completely re-established (see Figure A1B). Drug-induced side effects persisted but decreased with the tapering of the drugs from 1 month onward (Figure A1C). From day 15 (T15) to around day 50 (T50) post MRT, the clinical score returned to baseline (1/5), as shown in Figure A1D. At the later timepoint, an episode of partial and convulsive seizures occurred in one dog, but they were rapidly controlled by prednisolone increase (clinical score 2/5 in that one dog). During the third month after MRT, the dogs presented neither neurologic nor unspecific symptoms, setting the clinical score back to baseline (1/5).
